# Integrative tracking methods elucidate the evolutionary dynamics of a migratory divide

**DOI:** 10.1002/ece3.1205

**Published:** 2014-08-21

**Authors:** Allison H Alvarado, Trevon L Fuller, Thomas B Smith

**Affiliations:** 1 Center for Tropical Research, Institute of the Environment and Sustainability, University of CaliforniaLa Kretz Hall, Suite 300, 619 Charles E. Young Dr. East, Los Angeles, California, 90095; 2 Department of Ecology and Evolutionary Biology, University of California621 Charles E. Young Dr. East, Los Angeles, California, 90095

**Keywords:** *Catharus guttatus*, divergence, geolocator, hybridization, migratory divide

## Abstract

Migratory divides, the boundary between adjacent bird populations that migrate in different directions, are of considerable interest to evolutionary biologists because of their alleged role in speciation of migratory birds. However, the small size of many passerines has traditionally limited the tools available to track populations and as a result, restricted our ability to study how reproductive isolation might occur across a divide. Here, we integrate multiple approaches by using genetic, geolocator, and morphological data to investigate a migratory divide in hermit thrushes (*Catharus guttatus*). First, high genetic divergence between migratory groups indicates the divide is a region of secondary contact between historically isolated populations. Second, despite low sample sizes, geolocators reveal dramatic differences in overwintering locations and migratory distance of individuals from either side of the divide. Third, a diagnostic genetic marker that proved useful for tracking a key population suggests a likely intermediate nonbreeding location of birds from the hybrid zone. This finding, combined with lower return rates from this region, is consistent with comparatively lower fitness of hybrids, which is possibly due to this intermediate migration pattern. We discuss our results in the context of reproductive isolating mechanisms associated with migration patterns that have long been hypothesized to promote divergence across migratory divides.

## Introduction

Why, when, and where birds migrate have been central questions in biology since the time of Aristotle (Alerstam 1990). Investigating divergent migratory behavior within a single species has been of particular interest because of its relevance to speciation (Irwin and Irwin 2005; Rohwer and Irwin 2011). Migratory divides represent the boundary between adjacent breeding populations whose migration pathways diverge, with individuals from each side traveling to distinct overwintering locations. Often falling along hybrid zones, migratory divides have been described as natural genetic laboratories (Bensch et al. 1999). Reconstructing a species' phylogenetic history can identify the origin of a migratory divide; however, a species' future evolutionary trajectory will be determined by present-day interactions between the two groups at the migratory divide. Therefore, understanding the evolutionary processes responsible for promoting divergence both in the past and present is fundamental to studying migration's potential role in speciation.

The intraspecific variation in migratory routes that characterize migratory divides may be the result of biogeographic history and/or regulated by existing ecological factors. Specifically, migratory divides may be an artifact of range expansion along postglacial colonization routes by populations that previously diverged in allopatry (Ruegg and Smith 2002; Ruegg et al. 2006) and/or may arise from divergent selection pressures for two optimal routes around a geographic barrier (Berthold et al. 1992; Bensch et al. 1999). The migratory divide in Swainson's thrush (*Catharus guttatus*) is clearly the product of range expansion and secondary contact following the divergence of isolated populations in Pleistocene refugia, indicated by strong concordance between the migratory divide and variation in neutral genetic markers (Ruegg and Smith 2002; Ruegg et al. 2006). Other species with migratory divides show little or no genetic structuring between migratory groups (Bensch et al. 1999; Perez-Tris et al. 2004; Davis et al. 2006). In these cases, it is unclear whether gene flow has eroded the signal of historical isolation or whether different migration patterns evolved relatively recently *in situ* as a product of strong divergent selection.

As there is potential for interbreeding and thus homogenization across migratory divides, a fundamental question is how do the two migratory forms remain distinct? At least two intriguing mechanisms have been hypothesized to maintain variation across migratory divides. First, as the two forms migrate to and from separate overwintering locations, spring arrival times may differ, potentially leading to asynchronous breeding and premating isolation (Bearhop et al. 2005; Rolshausen et al. 2010; Ruegg et al. 2012; but see Rolshausen et al. 2013). Second, if some mating and gene flow does occur between the two migratory groups, reduced fitness of hybrids resulting from an intermediate migratory direction could maintain variation by leading to a stable cline at the hybrid zone (Barton and Hewitt 1989). Ultimately, a combination of these and other reproductive isolating mechanisms may further promote population divergence and ultimately drive speciation across a migratory divide (Helbig 1991a,b; Berthold et al. 1992; Rolshausen et al. 2009).

A key challenge to investigating these evolutionary processes has been the limited ability to track individual songbirds throughout the annual cycle (Webster et al. 2002; Bowlin et al. 2010; Bridge et al. 2011). Documenting long-distance movements during migration has traditionally been difficult in passerines because of their small size, which prevents the use of GPS tags. Furthermore, band-recapture rates of small birds are typically low (Webster et al. 2002), necessitating that migratory movements be studied with indirect methods including the use of distinguishing phenotypic traits, genetic markers, and/or isotopes (Bensch et al. 1999; Chamberlain et al. 2000; Ruegg and Smith 2002; Kelly et al. 2005). Although these indirect methods have been productive for identifying broad-scale patterns of migratory connectivity between breeding and nonbreeding (i.e., migrating and overwintering) locations, it is rare to uncover a diagnostic signature linking specific populations at a fine spatial scale. However, an important addition to the toolbox for studying songbird migration are geolocators, or data loggers that use variation in light levels to track an organism's year-round movements (Stutchbury et al. 2009; Cormier et al. 2012; Delmore et al. 2012). The ongoing weight reduction in geolocator devices now allows their use on small songbirds, permitting us to determine their migration routes and overwintering destinations with increased precision (Fox 2010; Bridge et al. 2011).

The goal of our study was to test what historical and contemporary evolutionary processes create and maintain migratory divides in North America, and to put these findings in the context of important reproductive isolating mechanisms potentially driving speciation in migratory birds. Our specific objectives were as follows: (1) to characterize a previously undescribed migratory divide in hermit thrushes (*Catharus guttatus*), a North American migratory songbird, by measuring genetic and phenotypic differentiation among populations at different spatial scales, (2) to establish the divergence times between the two migratory groups and thus, identify whether this migratory divide originated as a result of secondary contact between formerly allopatric populations, (3) to estimate migratory distance, migratory direction, and overwintering locations of individuals breeding east and west of the divide using two complementary methods: broad-scale tracking using genetic markers and fine-scale tracking using geolocator technology, and (4) to determine whether individuals from the hybrid zone at the center of the divide have an intermediate migratory pattern and lower return rates, potentially indicating lower fitness.

## Materials and Methods

### Study species

**Figure 1:**
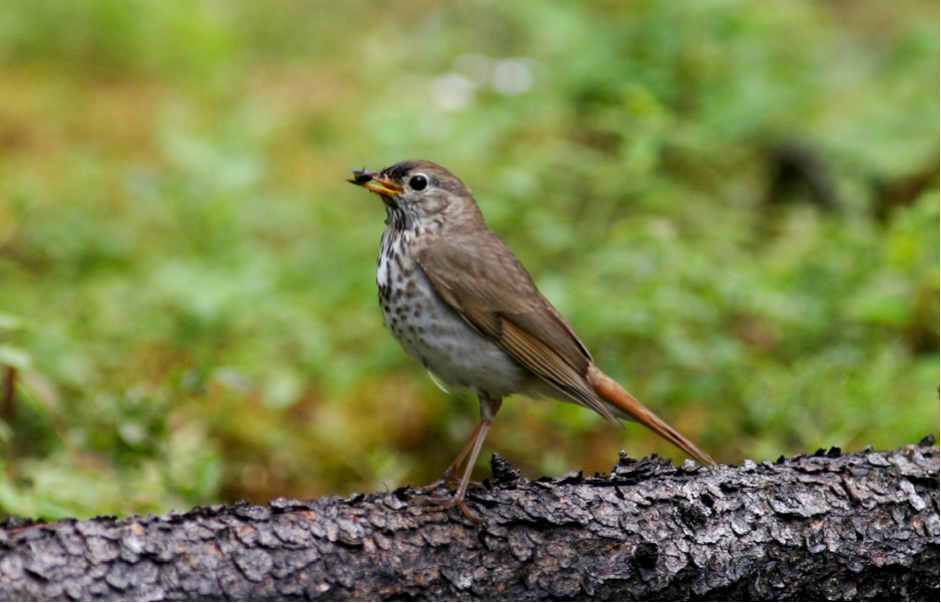
Hermit thrush (*Catharus guttatus*) on the breeding grounds in British Columbia, Canada. Photo by Robert McMorran.

Hermit thrushes (Fig.1) have an extensive range, breeding throughout most of North America in coniferous, deciduous, and mixed forests (Dellinger et al. 2012). Intraspecific patterns of phenotypic variation are complex, with as many as 12 subspecies described (Dellinger et al. 2012). These subspecies are divided into major groups with geographic ranges that mirror many other avian taxa (Weir and Schluter 2004), making the hermit thrush an ideal candidate for studying the evolutionary processes which dictate biogeographic patterns of avian diversity throughout North America. Hermit thrushes overwinter throughout the southern United States and Mexico; however, little is known about the specific overwintering destinations of particular breeding populations or their migratory routes (Dellinger et al. 2012).

### Geographic sampling

We sampled hermit thrush populations at three different geographic scales. First, we focus on seven breeding populations spanning from the west coast to the east coast across the breeding range. Using this dataset, we examine range-wide genetic variation at six microsatellite loci (*n* = 74) and mitochondrial DNA sequence variation in ATP-synthase 6 and 8 (ATPase) (*n* = 71). The seven sampling locations are peninsular Alaska, central Alaska, western British Columbia, eastern British Columbia, Manitoba, Michigan, and Connecticut.

**Figure 2:**
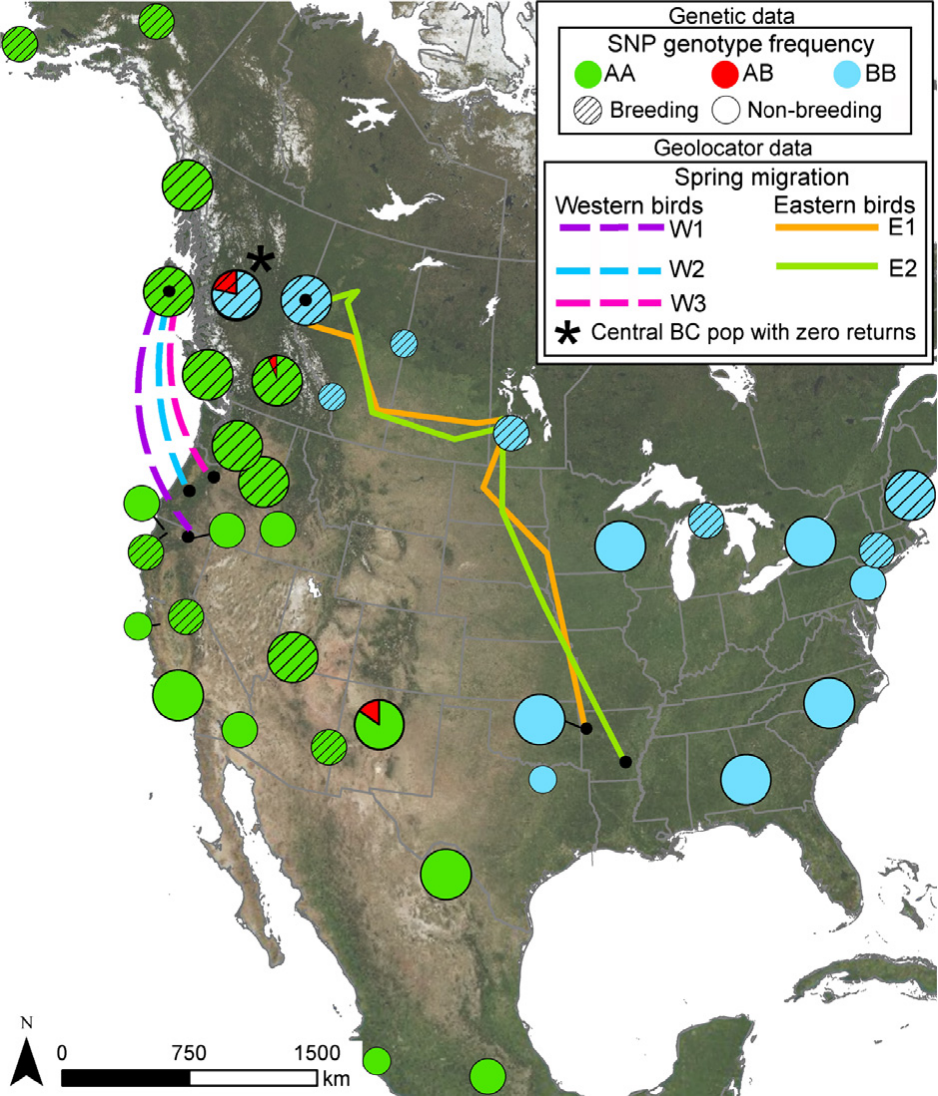
Geolocators and range-wide genetic data reveal a migratory divide in hermit thrushes. Geolocators indicate western British Columbia (BC) birds (*n* = 3) overwinter in California and Oregon (W1–W3) while eastern BC birds (*n* = 2) overwinter in Arkansas (E1–E2). Solid lines represent spring migration routes of eastern BC birds. Dotted lines link breeding and overwintering locations of western BC birds. Black dots connecting lines represent geolocator deployment/retrieval sites on breeding grounds and the centroid of overwintering home range on nonbreeding grounds. For our range-wide sample of birds (*n* = 380), we genotyped a *β*-fibint7 SNP within breeding (hatched circles) and nonbreeding (nonhatched circled) populations. Circle diameter represents genotype frequency (small circles = 1–3 birds; medium circles = 4–10 birds; large circles =11–20 birds; also see Table S1 for sample sizes). On the breeding grounds, genotypes AA (green) and BB (blue) occur west and east of the migratory divide in BC, respectively. During the nonbreeding season, these genotypes do not mix, indicating birds from opposing sides of the divide migrate different directions and are geographically separated while overwintering. Heterozygote genotype AB (red) is only found in central BC during the breeding season and only in New Mexico during the nonbreeding season, suggesting a likely intermediate nonbreeding location for birds from the hybrid zone.

Second, we focus on a much larger dataset that includes 13 more breeding populations (in addition to the seven listed above) and 17 nonbreeding populations (See Fig.2 and Table S1 for all location names and geographic coordinates). The purpose of this extensive sampling scheme was to establish the existence of a migratory divide in this species, to pinpoint its location on the breeding grounds, and to evaluate broad-scale patterns of migratory connectivity (Webster et al. 2002) between breeding and nonbreeding populations. Using this dataset, we genotyped a total of 380 individuals, including 208 birds from a total of 20 breeding populations and 172 birds from a total of 17 nonbreeding populations (i.e., migratory stopover and overwintering locations), for a single nucleotide polymorphism (SNP) from beta-fibrinogen intron 7 (*β*-fibint7). Sample sizes for each of the 37 populations can be found in the Supporting Information (Table S1).

Third, we focus on the migratory divide by examining three populations in British Columbia (BC), i.e., western, central, and eastern BC. According to our SNP analysis described above, this area represents the contact zone between the divergent migratory groups. Here, we attempt to track year-round spatial movements of individuals from each of the three populations by attaching geolocators, which require recapture of birds the following year to download spatial data. As no tagged birds returned to the central BC site, we only include migration route and overwintering location for individuals returning to western and eastern BC. However, we do include morphological data and return rates for all three populations.

### Laboratory techniques and analyses

We extracted genomic DNA from blood or feather samples using a Qiagen Extraction Kit (Valencia, CA, USA). We screened individuals for genetic variation at six microsatellite loci (Gibbs et al. 1999; Clegg et al. 2003): Cu*μ*02, Cu*μ*04, Cu*μ*10, Cu*μ*28, Cu*μ*32, and WpD30. Forward primers were tagged with a fluorescent dye (universal M13 forward primer). We set up reactions using a multiplex PCR kit (Qiagen Inc. Venlo, Limburg, Netherlands), ran PCR products on an ABI3730 capillary sequencer (Applied Biosystems), and scored alleles using GeneMapper software Foster City, California, USA (Applied Biosystems).

We tested for linkage disequilibrium (LD) and deviations from Hardy–Weinberg equilibrium (HWE) using GENEPOP 4.0 (Raymond and Rousset 1995) and applied sequential Bonferroni correction (Rice 1989). The program STRUCTURE version 2.3.2 (Pritchard et al. 2000) was used to evaluate the level of population structure. Five independent runs with a burn-in of 100,000 followed by 10^6^ iterations were performed using the admixture model and location prior function for *K* = 2–7. We then used the Evanno method (Evanno et al. 2005) to determine the appropriate *K* value.

Additionally, we amplified a 571 base pair (bp) fragment of ATPase using primers CO2GQL and CO3HMH (Eberhard and Bermingham 2004). Samples were sequenced on an ABI 3730 automated sequencer. We used SEQUENCHER 4.2.2 (GeneCodes) to automatically align sequences and checked each variable site visually for accuracy. We constructed a minimum-spanning network from absolute distance values between mtDNA haplotypes of ATPase using ARLEQUIN 3.1 (Excoffier and Smouse 1994). We also used ARLEQUIN to estimate population structure of ATPase by conducting analysis of molecular variance (AMOVA) and tested for significance via nonparametric permutation methods (Excoffier et al. 2005). Sampling sites were grouped to maximize among-group variance (*ϕ*_CT_). The two groups of populations identified based on mitochondrial differences were consistent with those based on analysis of microsatellite variation.

We used IMa to estimate divergence times between two major mitochondrial haplogroups, using the full model, which estimates six parameters including migration. All our initial analyses were run with large, flat priors to determine the limits that encompassed the entire posterior distribution for different parameters. We ran each final analysis for at least 12 million steps after a burn-in of 1 million steps using 10 chains. We used a geometric heating scheme with heating parameters set to g1 = 0.8 and g2 = 0.8. Mixing of the chains was monitored by observing effective sample sizes (ESS) and inspecting parameter plots for trends. In all our analyses, the ESS value for each parameter was >100 (Hey 2007), and results were verified with a replicate run using a different starting seed. We included an inheritance scalar (0.25) and mutation rate (1.05 × 10^−8^ substitutions per site per year) (Weir and Schluter 2008) in the data files to scale the parameter estimates to demographic units.

To identify a SNP that differentiated individuals on either side of the migratory divide, we first used PCR to amplify a 853 base pair (bp) fragment of beta-fibrinogen intron 7 (*β*-fibint7) in 25 individuals using primers FIB-B17U and FIB-B17L (Prychitko and Moore 1997). After sequencing on an ABI 3730 automated sequencer and aligning sequences automatically using SEQUENCHER, we designed primers using Primer3 software (forward: 5′-TGAAGCAGCTAAGAAAAACAAA-3′ and reverse: 5′-TCAATCAGTAAACCAAAATGACA-3′ Whitehead Institute, Cambridge, Massachusetts, USA) to target a 28 bp fragment (or amplicon) that included the diagnostic SNP differentiating groups on either side of the migratory divide. Real-time PCR and high-resolution melting (HRM) (Supporting Information) were performed on a LightCycler®480 instrument (Roche Diagnostics, Mannheim, Germany) twice for each sample.

The HRM analysis was performed using Gene Scanning Software Version 1.5.0 (Roche Diagnostics), allowing clustering of samples into groups based on difference plots obtained from melting curve shapes. To guarantee a high degree of detection sensitivity, HRM analysis sensitivity settings were 0.7 (high sensitivity). HRM results were confirmed through sequencing all heterozygote genotypes (*n* = 6) and a random subset of samples (*n* = 25) in addition to those used for screening (*n* = 25), which served as positive controls. To visualize frequency differences of *β*-fibint7 genotypes among populations across the continent, we mapped frequencies of homozygotes and heterozygotes from breeding and nonbreeding sites throughout North America.

### Morphological measurements and analysis

We assessed morphological variation of 35 adult male hermit thrushes from two populations (western and eastern BC), one on each side of the migratory divide. Using principal components analysis (PCA), we analyzed wing length, Kipps' index (a measure of the distance between the longest primary feather and the longest secondary feather on a folded wing) (Lockwood et al. 1998), and five additional morphological traits (tail length, tarsus length, bill length, bill width, and bill depth). To determine whether western and eastern BC populations were morphologically distinct, we conducted t-tests on PC1 and PC2.

To investigate whether western and eastern BC populations varied in morphological traits specifically related to flight efficiency, we focused on two morphological traits, i.e., wing length and Kipps' index, representing size and shape parameters, respectively. Kipps' index has been described as the best wingtip shape index for measuring overall proportions of the wing (Lockwood et al. 1998). We conducted a t-test on each variable to identify significant differences between the two groups. Because eastern BC birds are larger in many traits, we also investigated whether populations differed in wing length and Kipps' index after adjusting for differences in overall body size using a generalized linear model (GLM) with PC1 (body size) as a covariate (Supporting Information and Fig. S1). In addition to body size, population (western, central, or eastern BC) was included as a factor in the GLM following established protocols (Mila et al. 2008; Berner 2011). The rationale for including the population adjustment was to differentiate the association between population and body size within populations from the effect among populations.

Finally, we wanted to determine whether birds from central BC were intermediate in their morphology, indicating a potential hybrid zone between the two migratory forms. After including morphological data on birds from central BC, we ran ANOVAs on PC1 and PC2 between all three populations (*n* = 48) and performed post hoc tests to identify significant differences between groups.

### Geolocator deployment and recovery

In June and July of 2009, we fitted 58 breeding adult males at three sites across the migratory divide in BC with a MK10S light-level geolocator (1.2 g; British Antarctic Survey) using a leg-loop harness made of kevlar thread (size 600, Cambridge, UK, Thread Exchange, NC). We deployed 17, 24, and 17 geolocators at sites in western BC (53.128 N, 131.707 W), central BC (54.252 N, 126.093 W), and eastern BC (55.517 N, 120.083 W), respectively. The western and eastern BC sites were selected to represent locations on either side of the migratory divide, while the central BC site was selected to represent the hybrid zone between the two migratory forms. The percent average body weight of the geolocator attachment was highest (4.86%) in western BC (where the birds are smallest), lowest (4.02%) in eastern BC (where the birds are largest), and intermediate (4.29%) in central BC (where birds are intermediate in size).

In June and July of 2010, we returned to the original capture sites, where we conducted exhaustive searching of all pre-existing and adjacent territories (Supporting Information). Five hermit thrushes returned with intact geolocators, which were removed upon recapture. Data were downloaded successfully from all geolocators retrieved. Recaptured birds returning in 2010 with geolocators were in good physical condition, with a very small average change in body mass (−0.32 g, or approximately 1% of the body weight). There was no significant difference between years (*t* = 0.804; df = 4; *P* = 0.47); however, a power analysis (*α* = 0.05, and power = 0.9) indicates that 2.35 g is the minimal difference in means able to be resolved with a sample size of five individuals.

Of the five birds returning with geolocators, three were from western BC, two were from eastern BC, and none were from central BC (Supporting Information). We conducted a Chi-square test to determine whether return rates on either side of the divide were similar enough to combine, followed by a McNemar's test to determine whether geolocator recovery rates were the same in the hybrid zone and on the east and west sides of the divide. We also ran a separate set of analyses to compare return rates between these populations with a capture–mark–recapture model in the MARK software package (White and Burnham 1999), which estimates an error around the rates in each area. We present error data under both scenarios (i.e., for the western and eastern BC locations combined and separate).

### Analysis of geolocator data: estimating nonbreeding location

Light-level geolocators are used to calculate spatial position based on ambient light-level readings with reference to time (Fox 2010). These devices measure the amount of light every 60 s and record the maximum value within each 10-min interval, recording transitions from day to night and vice versa (Supporting Information). Latitude estimates are based on day length, while longitude estimates are based on the timing of recorded midday or midnight.

For our analysis, we focused on two time periods: (1) overwintering and (2) spring migration. We derived the overwintering locations for each individual based on latitude and longitude location estimates during an 85-day period (November 7, 2009 to January 31, 2010), during which time point localities were very stable. We calculated each bird's home range using kernel density estimation with the Hawth's Tools extension in ArcGIS 10.1 (ESRI, Redlands, CA, USA). This provides a probabilistic model of the area that the bird uses as its home range with 95% confidence (for a discussion of the statistical properties of kernel methods, see Worton 1989). The width of the home range was estimated from the data using least squares cross validation with the adehabitat library in the R software package. Next, we constructed a path linking the centroid of each bird's overwintering home range to its known breeding location. This analysis used the Convert Locations to Paths function in Hawth's Tools.

The timing of spring migration for birds returning to eastern BC (i.e., mid-April) made it possible to reconstruct their spring migration routes using both latitude and longitude (solid lines in Fig.2). However, the timing of spring migration for birds from western BC was earlier and overlapped with the spring equinox, during which latitude cannot be determined precisely (Supporting Information). Therefore, in Fig.2, we connect dotted lines between the breeding location and centroid of wintering point localities for each western BC bird because the exact spring migration routes could not be reconstructed.

## Results

### Genetic divergence across the breeding range

Microsatellite markers and mitochondrial DNA showed significant levels of genetic differentiation (with Bayesian analysis identifying two main groups) among the seven sampling locations spanning from the west coast to the east coast across the breeding grounds.

**Figure 3:**
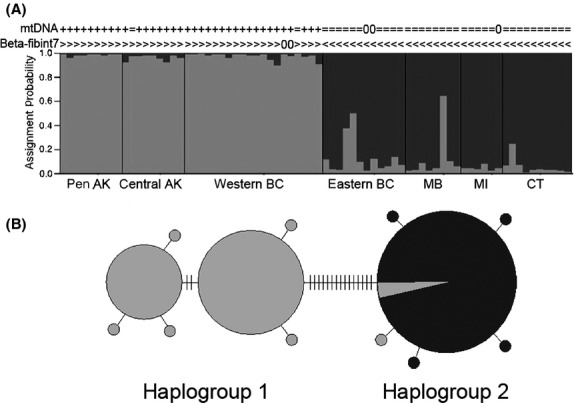
Genetic results showing differentiation among seven hermit thrush populations. Sampling locations occur from west to east across the breeding range: peninsular and central Alaska (AK), western and eastern British Columbia (BC), Manitoba (MB), Michigan (MI), and Connecticut (CT) with the following sample sizes: *n =* 9, 9, 20, 12, 8, 6, and 10, respectively. (A) Probability of assignment for 74 individuals to two genetic clusters based on variation at six microsatellite loci. Western and eastern BC populations show high assignment probability to different clusters, despite close geographic proximity. Symbols above the structure plot indicate mtDNA haplotype (+ and = represent haplogroups 1 and 2, respectively) and *β*-fibint7 genotype (> and < represent genotypes AA and BB, respectively). The symbol 0 denotes no data for that individual. (B) Minimum-spanning network of 13 mitochondrial DNA haplotypes of ATPase (571 base pairs) distributed among 71 individuals. Gray shading denotes individuals from peninsular and central AK, and western BC; black shading denotes individuals from eastern BC, Manitoba, Michigan, and Connecticut. Overall, there is considerable geographic structuring, with 15 base pair changes separating the two haplogroups.

There was no evidence of LD or departure from HWE across populations for any of the microsatellite loci after Bonferroni correction. Likelihood values reached a maximum at *K* = 2, and at this level Bayesian analysis of microsatellite data showed strong population structure which segregated geographically (Fig.3A). The two Alaska sites and western BC show strong differentiation from eastern BC, Manitoba, Michigan, and Connecticut. The most striking pattern is that the western BC and eastern BC populations generally have high assignment probability to different clusters, despite their close geographic proximity.

Consistent with the microsatellites, we also found high sequence divergence in mitochondrial DNA between the two groupings of populations identified by Bayesian clustering (df = 1; *ϕ*_CT_ = 6.47 = 87%; *P* < 0.03; Table S2). Sequencing 71 individuals for ATPase (571 bp) revealed 13 unique haplotypes that cluster into two major haplogroups (Fig.3B), which are separated by 15 base pair changes and have pairwise sequence divergence of 2.6%. IMa analysis indicates the two major haplogroups diverged about 960,000 years before present (ybp) (*t* = 6.0) with 95% high posterior density credibility of 436,000–1,387,000 ybp. The haplotype network (Fig.3B) indicates considerable geographic structuring. All birds from eastern BC, Manitoba, Michigan, and Connecticut belong to haplogroup 2. Conversely, almost all individuals from the two Alaska sites and western BC belong to haplogroup 1.

We did uncover a few mismatches in assignment when comparing different genetic markers (Fig.3). We identified two individuals breeding west of the divide that had mismatched genotypes. Specifically, one bird from central Alaska and one bird from western BC had mitochondrial haplotypes from haplogroup 2 (typical of populations east of the divide), but they otherwise had microsatellite and *β*-fibint7 genotypes typical of populations west of the divide (Fig.3A). We also identified three individuals breeding east of the divide that were potentially mismatched. Specifically, two birds from eastern BC and one from Manitoba have high assignment probability (>40%) to the microsatellite cluster west of the divide (Fig.3A). This indicates a likely mismatch because all three individuals otherwise had mitochondrial and *β*-fibint7 genotypes typical of populations east of the divide.

### Patterns of migratory connectivity using genetic data

The SNP in *β*-fibint7 differentiated populations on either side of the divide in BC. Among 50 sequences (obtained from 25 individuals), only two of 853 positions were variable, identifying three unique alleles. Although *β*-fibint7 showed lower sequence divergence than ATPase, this marker was highly informative because variation at position 619 reveals strict geographic structuring and the location of the migratory divide (Fig.2). Genotyping 380 individuals from both the breeding and nonbreeding areas for variation at this position revealed 232 homozygotes for cytosine (C) (genotype AA), 142 homozygotes for guanine (G) (genotype BB), and 6 heterozygotes (genotype AB).

All individuals breeding west of the divide (throughout Alaska, western BC, and the contiguous states of the western US) have genotype AA, while all individuals breeding east of the divide (occurring throughout eastern BC, central Canada, and the eastern US) have genotype BB. On the breeding grounds, the occurrence of heterozygotes is limited to two populations in central BC.

During the nonbreeding season, there continues to be strong geographic segregation between alleles; genotype AA occurs only in the western United States and Mexico, while genotype BB is primarily found in states east of Texas. On the nonbreeding grounds, heterozygotes are only found in New Mexico.

### Geolocator recovery, overwintering location, and migratory distance across the divide

At the beginning of the 2010 breeding season, five of 58 individuals returned to their original sampling locations on the breeding grounds of BC with their geolocators attached (*n* = 2 from eastern BC, and *n* = 3 from western BC). Data from these loggers revealed the two eastern BC birds overwintered in Arkansas (southeast of their breeding location), while the three western BC birds overwintered in northern California and Oregon (south of their breeding location) (Fig.2).

As a result of their disparate overwintering locations, the distance traveled by birds on either side of the migratory divide differed as well. The Euclidean distance between breeding and overwintering locations for the two birds east of the divide (2887 and 3110 km) was over twice that of the three birds west of the divide (1171, 1174, and 1153 km). Variation in migratory direction and distance results in strong segregation between these birds from eastern and western BC during the nonbreeding season.

### Variation in morphology

**Figure 4:**
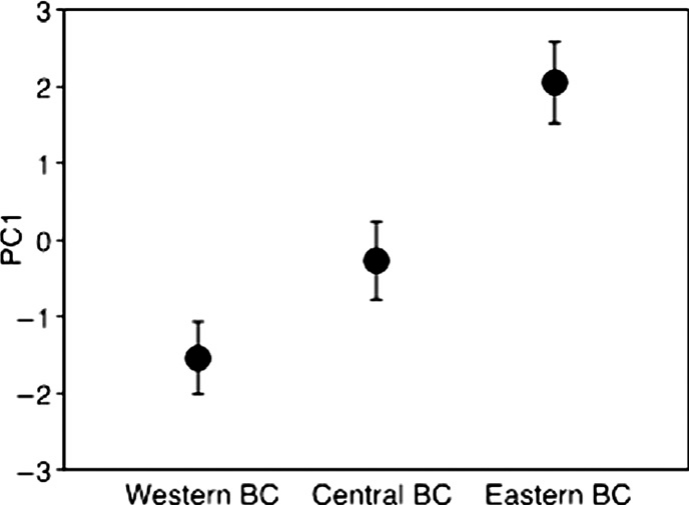
Morphological variation among populations from western, central, and eastern British Columbia (BC). The three populations differed significantly in their PC1 values (*F*_2,45_ = 62.43; *P* < 0.0001). PC1 explains 51.6% of the variation and includes the following morphological traits (wing length, Kipps' index, tail length, tarsus length, bill length, bill depth, and bill height). Post hoc comparisons indicate all three populations are significantly different from one another in their PC1 values at a significance level of *P* = 0.002 or below. The intermediacy of values in central BC suggests this is a hybrid zone.

Populations from western and eastern BC are morphologically distinct (Fig.4). Factor loadings from PCA of seven morphological traits revealed that the first principal component (PC1) explains 51.6% of the total variance and generally describes size (positive loading on all variables) (Table S3). The second principal component (PC2) explains only 16.2% of the total variance. Populations from eastern and western BC are significantly different with respect to PC1 (*t* = −10.53; df = 33; *P* < 0.001), but not PC2 (*t* = 0.018; df = 33; *P* = 0.986). Both wing length (*t* = −11.5; df = 33; *P* < 0.001) and Kipps' index which reflects wing pointedness (*t* = −2.4; df = 33; *P* = 0.02) are significantly greater in birds from eastern BC (Fig. S2). After the size-adjustment, the trend of relatively longer wing length and a more pointed Kipp's index continues for eastern BC birds, although the difference is no longer significant for size-adjusted Kipps' index (*P* = 0.058) (Supporting Information).

Birds from central BC are intermediate in size relative to those from western and eastern BC (Fig.4 and Fig. S3). The three populations significantly differ in their PC1 values (*F*_2,45_ = 62.43; *P* < 0.0001), and post hoc comparisons reveal that all three populations are significantly different from one another at a significance level of *P* = 0.002 or below. There are no significant differences between populations in their PC2 values (*F*_2,45_ = 0.14; *P* = 0.86).

### Intermediate migration pattern and reduced fitness at the hybrid zone

Based on our SNP analysis of 213 breeding birds across 20 sampling sites, central BC is the only breeding location where heterozygote individuals for *β*-fibint7 (genotype AB, *n* = 4) occur (Fig.2). This genetic data combined with an intermediate morphology (Fig.4 and Fig. S3) indicate central BC represents a hybrid zone (Berthold and Querner 1982) between the two migratory forms on the breeding grounds. During the nonbreeding season, despite extensive sampling (172 individuals across 17 nonbreeding sampling sites), the only location where we found heterozygote individuals (AB genotype, *n* = 2) was in New Mexico (Fig.2), suggesting an intermediate nonbreeding location for birds migrating from the hybrid zone.

Although we attached geolocators to birds at the hybrid zone in the center of the migratory divide, no birds returned to this area the following year. We combined the eastern and western BC return rates because there was no significant difference between them (*χ*^2^ = 0.235; df = 1; *P* = 0.6282). The recovery rate in the contact zone was significantly lower than on either side of the divide (McNemar's *χ*^2^ = 32.03; df = 1; *P* < 0.0001). Specifically, 14.7% of geolocators attached to birds east or west of the migratory divide were recovered the following year, whereas none were recovered at the contact zone (despite deploying a greater number of geolocators in 2009 and having greater search effort in central BC compared with either of the other locations in 2010, see Supporting Information). The capture–recapture model provided estimates of the error around these recovery rates. In the hybrid zone, the recovery rate was 0% with an error of 5.33%. When eastern and western BC are combined, the recovery rate is 14.7% in the combined area with a lower limit of 10.35% and an upper limit of 19.06%. Thus, the recovery rate in the hybrid zone (upper limit 5%) is significantly lower than areas away from the divide. Furthermore, when we look at the error around eastern and western BC separately, there is still no overlap between the error in the hybrid zone and either side. In western BC, the rate was 17.65% with an error of 6.06%, and in eastern BC, the rate was 11.76% with an error of 6.33%.

## Discussion

In this study, we investigate historical and contemporary evolutionary processes that have long been hypothesized to create and maintain migratory divides, potentially promoting further divergence and ultimately driving speciation in some migratory birds. By combining data from genetics, geolocators, and morphology, we identify the location where two highly divergent lineages of hermit thrushes come into geographic contact in BC, we establish rangewide patterns of migratory connectivity between breeding and nonbreeding populations, and we assess how differences in migration patterns across the migratory divide may promote some forms of reproductive isolation.

### Genetic divergence: the origin of the migratory divide

Our results indicate that periods of isolation followed by secondary contact initially shaped patterns of diversity across the range of the hermit thrush. Our estimates based on ATPase indicate that the major splitting event (between haplogroups 1 and 2) occurred approximately 0.96 million years before present (Mybp). During the Early to Mid-Pleistocene (1.8 – 0.8 Mybp), major ice sheets united into a single mass, fragmenting the boreal zone of North America into eastern and western sectors for prolonged periods (Barendregt and Irving 1998), likely causing high divergence between populations on either side of the migratory divide. To our knowledge, the level of intraspecific genetic divergence we identified between hermit thrush lineages is among the highest observed across a migratory divide to date (Irwin et al. 2001; Ruegg and Smith 2002; Perez-Tris et al. 2004; Irwin and Irwin 2005; Mettler et al. 2013; Rolshausen et al. 2013). The high degree of divergence among hermit thrush lineages is more consistent with several co-distributed taxa called “superspecies complexes” occupying North America's forests (Weir and Schluter 2004). Many of these “superspecies complexes” are monophyletic groups of sister taxa that have recently been elevated to full species status (Amadon 1968; Chesser et al. 2010).

In cases where migratory divides have resulted from secondary contact following periods of isolation (Ruegg and Smith 2002), divergent populations of migrating birds are thought to be retracing their ancestors' routes of range expansion after the last glacial maximum (Ruegg and Smith 2002; Ruegg et al. 2006). Why do these species maintain the same migratory routes for thousands of years while other species evolve new migratory paths quickly, in some cases over just a few decades (Berthold et al.^1992^)? Migratory direction is assumed to be under strong genetic control in passerines (Helbig 1991a, 1996; Pulido et al. 2001) and thus, is subject to drift and natural selection (Barton and Gale 1993). Because divergent selection and gene flow often work in opposition to each other (Barton and Hewitt 1985, Barton and Hewitt 1989), the interplay between these two forces likely dictates whether and how quickly migration routes can change.

Bayesian assignment tests of microsatellites indicate high assignment probability of populations on either side to different clusters, indicating there is likely a barrier to gene flow that is restricting homogenization across the divide (Harrison 1993). However, for five of 74 individuals, we did find evidence of mismatched genotypes, likely indicative of complex hybridization between populations spanning the migratory divide. Because contemporary patterns of gene flow dictate whether ancestral migration routes will be preserved or eroded over time (Ruegg and Smith 2002; Ruegg et al. 2006), our future research will use additional makers and sampling locations to quantify gene flow throughout this region at a fine spatial scale.

### Characterizing the divide: from migration to morphology

While mtDNA and microsatellites helped identify the origin of the migratory divide, the *β*-fibint7SNP was essential for establishing broad-scale patterns of migratory connectivity. We only find the western genotype AA in western nonbreeding sites and the eastern genotype BB in eastern nonbreeding sites (Fig.2). This indicates broad-scale patterns of migratory connectivity between breeding and nonbreeding locations, providing strong evidence of separation on the wintering grounds between groups east and west of the migratory divide.

The use of geolocators complemented this much larger genetic dataset by identifying nonbreeding locations of individuals from eastern and western BC populations at a finer-spatial scale. The two birds we tracked from eastern BC overwintered in Arkansas, while the three birds from western BC overwintered in Oregon and northern California. Although we need to use caution when making inferences based on these few geolocator returns, the geolocator dataset is nevertheless extremely valuable. First, we intend to incorporate geolocator data into our ongoing research on the strength of migratory connectivity in hermit thrushes using methods which combine genetic and isotopic data (Rundel et al. 2013). Second, these geolocator-derived locations provide a reference point for current and future band recoveries. Of the hundreds of hermit thrushes that we have banded among our BC breeding populations, to our knowledge only a single individual has been recovered on the wintering grounds (i.e., a male from the western BC site banded in June 2006 was recovered in northern California in 2009), and this overwintering location is consistent with our western BC geolocator returns.

Another valuable contribution of geolocator data, which complements our genetic and morphological data, are the migratory distance estimates they provide for birds from eastern and western BC. Despite low sample sizes, geolocators indicate the birds we monitored from eastern BC travel at least twice as far, when measured as the crow flies, to their overwintering grounds in Arkansas compared with their western counterparts migrating to Oregon and northern California. The actual difference in migration distance between the two migratory forms is likely greater because the eastern BC birds did not actually travel “as the crow flies” during their spring migration. Instead, they traveled northward from Arkansas to a stopover site in central Canada before migrating westward toward BC (Fig.2).

Sustained flight during migration is costly and migratory distance can exert selection on morphological traits associated with flight efficiency (Lockwood et al. 1998; Baldwin et al. 2010). We predicted that wing size (i.e., length) and pointedness (i.e., Kipps' index) would increase with migratory distance. Mechanistically, longer and more pointed wings likely function as an adaptation for migrating longer distances due to high aspect ratio; this generates more lift and reduces drag, thereby minimizing the cost of flight (Lockwood et al. 1998; Baldwin et al. 2010). As predicted, the populations east and west of the divide show morphological differences, including longer, more pointed wings in birds from eastern BC. When we adjust these variables for isometric effects of size, eastern BC birds still show longer more pointed wings, but the difference in Kipps' index is no longer significant (*P* = 0.058). Nevertheless, these data suggest that morphometric differences between birds east and west of the divide may be adaptive.

### The costs of an intermediate migratory pattern

We found genetic admixture as well as an intermediate morphology of individuals in central BC, indicating hybridization does occur in this region. The hybrid zone in central BC is the only breeding location where we found heterozygotes for the *β*-fibint7 SNP, despite extensive sampling across the breeding range (Fig.2). Furthermore, this diagnostic heterozygous genotype is only present at a single nonbreeding location. Among the 17 nonbreeding sites we screened, heterozygotes for the *β*-fibint7 SNP are only found to occur in New Mexico (Fig.2). This nonbreeding location is intermediate between the overwintering sites of populations on either side of the divide as indicated by geolocator data (i.e., birds from western BC overwinter in Oregon and northern California, while those from eastern BC overwinter in Arkansas). Only laboratory studies of European blackcaps (*Sylvia atricapilla*) in Emlen cages have shown an intermediate migratory direction in F1 offspring of mixed pairs from opposite sides of their migratory divide in Europe (Helbig 1991a). It is not surprising that an intermediate migratory direction has been difficult to detect in wild populations, likely because hybrids with intermediate migratory orientation may migrate to inappropriate sites where survival is poor (Berthold et al. 1992; Helbig 1991a,b; Bensch et al. 1999; Rolshausen et al. 2009; but see Veen et al. 2007). This is consistent with our significantly lower recovery rate of birds with geolocators at the center of the divide, suggesting individuals from the hybrid zone may be experiencing lower inter-annual survivorship compared with birds on either side of the divide. As we use return rates as a proxy for fitness, this supports the idea that an intermediate migratory pattern may serve as an important postzygotic isolating mechanism among the two hermit thrush lineages.

In this study, the integration of geolocator and genetic data allowed us to evaluate the overwintering destination of all three populations spanning the migratory divide. There are, however, limitations associated with both types of data. First, even though the SNP data provide evidence that birds from central BC have an intermediate nonbreeding location, we need to be cautious about inferring that their migratory direction or vector is also intermediate since we do not have data on their migration route. Second, while the *β*-fibint7 SNP does identify a nonbreeding location of heterzyogotes, we cannot use this marker to pinpoint specific nonbreeding locations of the homozygous individuals (genotypes AA and BB) within the contact zone. For example, it is unknown whether the pure AA genotypes mixed with heterozygotes in New Mexico also migrate from the vicinity of the hybrid zone in BC or other western breeding populations. The use of additional genetic markers to distinguish between these scenarios is necessary to resolve this. Third, we cannot exclude the possibility that our sampling methods caused lower return rates in central BC; however, we consider this scenario highly unlikely. We used the same exhaustive search protocol at all three locations (Supporting Information). Furthermore, there is no indication that the weight of the geolocators relative to body size influenced our results. If so, we would expect return rates to be lowest in western BC, where geolocators represent the highest average percent body weight. Instead, eastern and western BC, which represent extremes in percent body weight, had similar return rates. There may, however, be interesting interactions between migration distances and the relative weight of the geolocator that caused return rates in eastern and western BC to balance out. For example, in western BC, the cost associated with carrying the heaviest geolocators relative to size may be offset by shorter migration distances.

Potential interactions between migratory distance, wing morphology, and geographic features along migration routes may also impact return rates. Populations east and west of the divide are likely retracing their ancestors' migration routes (Ruegg et al. 2006), and their particular wing characteristics and other physiological adaptations have likely evolved to facilitate the journey. However, hybrids may not possess the physical traits required to successfully navigate a novel route. The deleterious effects of an intermediate migratory direction are evident in Europe where species with migratory divides typically circumvent the Alps or Mediterranean Sea during migration (Helbig 1991a,b; Bensch et al. 1999). Compared with the well-defined geographic barriers faced by European birds (Alerstam 2001; Irwin and Irwin 2005), less is known about potential barriers circumvented by North American species with migratory divides (Brewer 2000; Ruegg and Smith 2002; Irwin and Irwin 2005). In our study, *β*-fibint7 data suggest that at some hermit thrushes from the hybrid zone migrate from central BC to New Mexico. If they take a direct route, they would encounter obstacles such as the Great Basin Desert and/or Rocky Mountains along the way. While such negative selective pressures associated with an intermediate migratory pattern remain to be confirmed through additional study, these mechanisms could act to reinforce population divergence across the migratory divide.

### Alternative reproductive isolating mechanisms

There are other potential isolating mechanisms, both pre- and postzygotic (Irwin and Irwin 2005; Rohwer and Irwin 2011), that could be relevant to this system. Potential prezygotic isolating mechanisms across migratory divides include variation in song (Ruegg et al. 2012), microhabitat use (Rolshausen et al. 2013), and spring arrival times (Ruegg et al. 2012). Although geolocators recovered in eastern and western BC indicate some variation in arrival time across the hermit thrush migratory divide (unpublished data), we do not have arrival time data from the hybrid zone. However, assuming one migratory form arrives at the hybrid zone earlier to establish mating pairs before the other migratory form arrives, there could be asynchronous breeding and reduced opportunities for hybridization between the two groups (Bearhop et al. 2005; Rolshausen et al. 2010; Ruegg et al. 2012). Bearhop et al. (2005) found that two different migratory forms of European blackcaps mate assortatively in areas of sympatry as a result of arrival time differences. However, Rolshausen et al. (2010) found that this form of prezygotic isolation is likely incomplete and suggests that postzygotic mechanisms may also be required for ongoing divergence and ultimately, speciation.

In our study, we explore an important potential postzygotic isolating mechanism, i.e., reduced fitness of hybrids due to an intermediate migratory pattern, that has long been hypothesized to promote divergence across migratory divides. However, it is possible that there are also genetic incompatibilities between the groups from either side of the divide leading to an intrinsic reduction in survival and/or infertility of hybrids (Coyne and Orr 2004). Although this scenario is plausible considering hermit thrushes have relatively high intraspecific sequence divergence in mtDNA compared with other avian taxa in the region (Ruegg and Smith 2002; Weir and Schluter 2004), we know that reproductive isolation is not complete because we have identified individuals of hybrid origin. Furthermore, we recognize that the *β*-fibint7 SNP underestimates the number of hybrids in the contact zone, and the mismatched genotypes among microsatellites, mtDNA and *β*-fibint7 indicate potentially complex backcrosses in populations farther away from the contact zone. While further study of the hybrid zone is needed, it is likely that many of these reproductive isolating mechanisms are working in concert to maintain the genetic, behavioral, and phenotypic differences that we document across this migratory divide.

## Conclusions

Our study uses a combination of very different and complementary approaches to address interesting questions on the past and present evolutionary mechanisms thought to generate and maintain migratory divides. Deep divergence in mtDNA and microsatellites reveals that the hermit thrush migratory divide is the result of secondary contact following Pleistocene divergence of two lineages. A combination of fine-scale tracking with geolocators and broad-scale tracking using a diagnostic genetic marker indicate the lineages correspond to distinct eastern and western migratory forms. Evidence of genetic admixture and the intermediate morphology of individuals reveal evidence of hybridization at the secondary contact zone in central BC. Birds from this site appear to migrate to an intermediate nonbreeding location (based on genetic data) and experience lower return rates (based on geolocator data), potentially suggesting lower fitness. Therefore, postzygotic isolating mechanisms related to migratory patterns are likely involved in maintaining high levels of genetic divergence and significant morphological differences among hermit thrush populations from either side of the migratory divide. It is unclear whether and at what stage the distinct migratory forms of hermit thrushes are in the speciation process (Coyne and Orr 2004; Irwin and Irwin 2005). However, the biogeographic histories (Weir and Schluter 2004) and patterns of divergent migratory behavior (Brewer 2000) of numerous other parapatric sister taxa, many of which have recently been designated as separate species, are similar to those of hermit thrushes. The mechanisms that historically create, currently maintain, and further promote population differences across migratory divides may also shape patterns of biodiversity and speciation in avian taxa of North America that have not traditionally been studied within this context.

## Data Accessibility

Data are available from the Dryad Digital Repository.

## Supporting Information

**Appendix S1.** Supporting methods and results. **Figure S1.** Workflow for size-adjustment of wing length and Kipp's index of wing pointedness. **Figure S2.** Average wing length and Kipps' index for western and eastern British Columbia (BC) populations. **Figure S3.** Jittered strip chart of morphometric measurements. **Table S1.** Coordinates and sample sizes at the 37 sampling locations for *β*-fibint7 SNP shown in Fig. 2 of the main text. **Table S2.** Analysis of molecular variance (AMOVA) for mitochondrial sequence variation. **Table S3.** Factor loading for morphological traits and derived principal components.
