# Supplementary material for: Integrative tracking methods elucidate the evolutionary dynamics of a migratory divide
Source: Ecol Evol. 2014 Aug 21;4(17):3456–69. doi: 10.1002/ece3.1205 (PMC4228619; doi:10.1002/ece3.1205)

**Integrative tracking methods elucidate the evolutionary dynamics of a migratory divide**

A. H. ALVARADO, T. L. FULLER, and T. B. SMITH

**Supporting Information: Supplementary Methods and Results**

***Sampling***

Individual birds were captured using mist-nets, and blood and/or feather samples were collected prior to their release. Breeding birds were captured from late May through July, and non-breeding birds were captured from early October through April. Information on the age, sex, and reproductive condition of each bird was noted. Sample sizes for each of the 37 locations are summarized in Table S1.

***Genotyping beta-fibrinogen intron 7 single nucleotide polymorphism***

The reaction mixture contained 10-20 ng of genomic DNA, 2.5 mM MgCl2 (Roche Diagnostics, Mannheim, Germany), 10 pmol of each primer, 5 µl 2x LightCycler®480 High Resolution Master Mix (Roche Diagnostics, Mannheim, Germany), and PCR grade water with a total reaction volume of 10 µl. The PCR cycling conditions included an initial activation at 95°C for 10 min followed by 35 cycles of 95°C for 1 min, 60°C for 1 min, and 72°C for 1 min and a final extension at 72°C for 15 min. To execute high resolution melting, amplicons were denatured with an initial hold at 95°C for 5 min, renatured at 40°C for 1 min, and subjected to a melting profile from 75°C to 85°C with a ramping degree of 0.05°C and 25 signal acquisitions per degree.

***Morphological measurements***

Wing length represents the unflattened wing chord and Kipps’ index is a measure of the distance between the longest primary feather and the longest secondary feather on a folded wing. Kipps’ index has been described as the best wingtip shape index for measuring overall proportions of the wing (Lockwood *et al.* 1998). Both were measured to the nearest 0.5 mm with a standard wing ruler. Five additional morphological traits were measured to the nearest 0.1 mm with digital calipers: tail length (from the uropygial gland to the tip of the longest retrix), tarsus length (from the tibiotarsus joint to the distal end of the tarsometatarsus), bill length (from the anterior end of the nares to the tip of the upper mandible), and bill width and depth (at the anterior end of the nares).

***Size-adjusting measures of wing length and Kipps’ index***

Because birds from eastern BC are generally larger than those from western BC, we used a general linear model (GLM) (Fig. S1) to control for the effects of overall body size on certain traits (e.g. wing length and Kipps’ index). This generated size-adjusted values for each of these variables, whose means would be compared across each population. We first conducted principle components analysis (PCA) on the morphological traits, which yielded high factor loadings on the first principle component (PC1; Table S2). These values were then used to represent overall size and were included in the GLM as a covariate. We conducted separate GLMs for wing length and Kipps’ index, each time removing that trait from the PCA so it did not simultaneously serve as the dependent variable and as part of the covariate. For example, when calculating size-adjusted wing length, we did not include wing length in the PCA.

After adjusting traits for differences in overall body size to account for possible allometric effects, we found that size-adjusted wing length was significantly longer in birds from eastern BC (*F*_2,32_ = 109.55; *p* < 0.0001). When corrected for overall size, birds from eastern BC also had longer size-adjusted Kipps’ distance than birds from western BC, but the difference was no longer significant (*F*_2,32_ = 3.12; *p* = 0.058).

***Geolocator attachment***

Upon capture, each bird that had a geolocator attached was also fitted with an aluminum service band on the leg for future identification. Each geolocator was constructed with a 15 mm stalk angled at 30º which was positioned on the bird such that the light sensor at the end of the stalk was exposed (i.e. not covered by feathers) when wings were open and closed. Following release, all birds flew without difficulty and were subsequently monitored for several hours.

***Geolocator recovery***

Five birds returned to the breeding grounds in 2010 with their geolocators attached (three were from western BC, two were from eastern BC sites, and none were from central BC). Three additional birds returned without their geolocators attached (two were from western BC, one was from eastern BC, and none were from central BC). To locate birds previously tagged with geolocators in 2009, we returned to the original site of capture in 2010 and used song playback to attract the territorial male to the mist net. When a territorial male appeared, he was captured within the first few minutes of playback. In our experience, territorial hermit thrush males respond very aggressively toward the playback (singing back and displaying on nearby perches as well as approaching the playback speaker). We used the same procedure to attract and capture male hermit thrushes on all adjacent territories in 2010, whether or not we had captured and tagged a hermit thrush on that adjacent territory during the 2009 breeding season. In 2010, we detected a total of 116 territories (34, 45, and 37 in western, central, and eastern BC respectively). Our total search area was approximately 102 hectares. This ensured that we would encounter a tagged individual even if he had moved to a nearby territory in the area the following year. Our search radius from the original point of capture the previous year was a minimum 500 m (i.e. if it was one of our isolated territories or occurred along the edge of a group of territories the previous year). However, in most cases, we searched a much larger radius (up to 3 km) because many of the territories where we originally tagged birds the previous year were grouped and therefore the adjacent territories were already inspected. All birds recaptured in 2010 were re-sighted within 150 m of their original capture location, despite thorough searching of adjacent territories, suggesting high site fidelity by males. We followed this targeted approach (i.e. where every male on territory within the 102 hectare study area was netted) with a systematic survey of these and adjacent areas. We walked along line transects, stopping every 100 m where we, again, used song playback to attract any hermit thrushes in the immediate vicinity. We stayed at each stopping location for a minimum of five minutes. If and when a hermit thrush approached, we stayed until both surveyors had a clear visual of the hermit thrush’s back (where a geolocator stalk would be visible) to minimize the likelihood that our targeted approach missed any bird returning to the area with a geolocator attached.

The combination of our two different methods required that each territory be inspected at least twice, with many territories visited additional times when there was no sign of a territorial male or if the male was not be captured on our initial visit. We spent approximately 107, 112 and 215 man-hours searching in the western, eastern, and central BC locations, respectively (where 17, 17 and 24 geolocators had been attached to birds the previous year). We spent more total hours at the central BC location for two reasons: 1) we originally tagged more birds there resulting in more territories to cover, and 2) upon finding no returning birds with the targeted method, we monitored each transect more than once. As a result, the average number of hours spent searching for each geolocator was 6.29, 6.59, and 8.96 man-hours at the western, eastern, and central BC locations, respectively. By combining these two methods (i.e. targeted and systematic), we consider our searches to have been exhaustive. While walking our line transects, almost all of the hermit thrushes we encountered were the territorial males that had been captured previously. In the rare case that we encountered an unbanded hermit thrush using the systematic transect method, it was usually engaged in non-aggressive, foraging behavior and upon capture was identified as a female.

***Analysis of light data***

The program Bastrak (British Antarctic Survey) was used to correct for clock drift. Based on pre- and post-deployment calibration at known locations, we selected a light-level threshold of 8, which corresponded to an average sun elevation of -4°. Based on these parameters, sunrise and sunset times were determined using TransEdit (British Antarctic Survey), and two daily geographic positions were estimated with BirdTracker (British Antarctic Survey). Since hermit thrushes are nocturnal migrants (Dellinger *et al.* 2012), we used only noon locations and did not apply BirdTracker’s movement compensation option. Locations derived from curves with shading events around sunset and sunrise, or those that were clearly anomalous (e.g. requiring unrealistic flight speeds), were excluded from the analysis. A previous study which assessed the accuracy of geolocators calculated a mean error ± s.d. of 186 ± 114 km (Phillips *et al.* 2004). In our study, calibration with a control logger indicated that point localities inferred based on light levels were within 100 km of the true longitude and latitude calculated using GPS.

**References**

Dellinger, R, Bohall Wood P, Peter W. Jones P, Donovan T (2012) *Hermit Thrush (Catharus guttatus). The Birds of North America No. 261* American Ornithologists' Union, Philadelphia, Pennsylvania.

Lockwood R, Swaddle JP, Rayner JMV (1998) Avian wingtip shape reconsidered: wingtip shape indices and morphological adaptations to migration. *Journal of Avian Biology*, **29**, 273-292.

Phillips RA, Silk JRD, Croxall JP, Afanasyev V, Briggs DR (2004) Accuracy of geolocation estimates for flying seabirds. *Marine Ecology-Progress Series* **266**, 265-272.

**Fig S1.** Workflow for size-adjustment of wing length and Kipp’s index of wing pointedness.

**Fig. S2.** Average wing length and Kipp’s index for western and eastern British Columbia (BC) populations. Wing length is measured as the unflattened wing chord and Kipp’s index is a measure of wing pointedness represented by the distance between the longest primary feather and longest secondary feather on the folded wing. Wing length (*t* = -11.5; d.f. = 33; *p* <0.0001) and Kipp’s index (*t* = -2.4; d.f. = 33; *p* = 0.02) were significantly longer in eastern BC birds (error bars represent 95% confidence intervals). This result is consistent with the prediction that wing length and wing pointedness is positively correlated to migration distance.

**Fig. S3.** Jittered strip chart of morphometric measurements. The morphometric data are plotted along a line representing the first principal component (PC1). A random vertical jitter was added to separate coincident points. Birds from central BC birds have intermediate morphology compared to individuals from western and eastern BC.

**Table S1.** Coordinates and sample sizes at the 37 sampling locations for β-fibint7 SNP shown in Figure 2 of the main text. The symbol * in the table denotes locations of the seven populations included in the microsatellite and mtDNA datasets , while the symbol † denotes the locations of the three populations included in the geolocator and morphology datasets.

| **Season** | **Location** | **State/Province** | **Country** | **Latitude** | **Longitude** | **Samples** |
| --- | --- | --- | --- | --- | --- | --- |
| Breeding | Primrose Lake | Alberta | Canada | 54.729 | -110.062 | 1 |
|  | Sibbald Creek | Alberta | Canada | 51.036 | -115.026 | 1 |
|  | Queen Charlotte's Island/ “western BC” | British Columbia *† | Canada | 53.129 | -131.707 | 18 |
|  | Maxan Lake/ “central BC” | British Columbia † | Canada | 54.252 | -126.093 | 14 |
|  | Swan Lake/ “eastern BC” | British Columbia *† | Canada | 55.517 | -120.083 | 12 |
|  | Mount Washington, Vancouver | British Columbia | Canada | 49.763 | -125.319 | 13 |
|  | Kamloops | British Columbia | Canada | 50.921 | -119.850 | 15 |
|  | Riding Mountain National Park/ “MB” | Manitoba * | Canada | 50.773 | -99.660 | 8 |
|  | Chatham | Alaska | USA | 57.989 | -135.397 | 14 |
|  | Denali/ “central AK” | Alaska * | USA | 63.594 | -149.611 | 10 |
|  | Mother Goose Lake/ “peninsular AK” | Alaska * | USA | 57.183 | -157.283 | 9 |
|  | Apache National Forest | Arizona | USA | 33.593 | -109.362 | 10 |
|  | Six Rivers National Forest | California | USA | 40.957 | -123.485 | 6 |
|  | Yosemite | California | USA | 37.795 | -119.864 | 10 |
|  | Bog Meadow Pond/ “CT” | Connecticut * | USA | 41.850 | -73.456 | 10 |
|  | Douglas Lake/ “MI” | Michigan * | USA | 45.579 | -84.742 | 6 |
|  | Brunswick Naval Air Station | New York | USA | 43.871 | -69.942 | 12 |
|  | Umatilla National Forest | Oregon | USA | 45.679 | -118.115 | 12 |
|  | Dixie National Forest | Utah | USA | 37.529 | -112.753 | 12 |
|  | Wenatchee National Forest | Washington | USA | 46.962 | -120.925 | 15 |
| Migrant | Múzquiz | Coahuila | Mexico | 28.383 | -101.851 | 12 |
|  | Morelos | Jalisco | Mexico | 19.586 | -104.275 | 2 |
|  | Boise | Idaho | USA | 43.605 | -116.060 | 8 |
|  | Hopewell | New Jersey | USA | 40.413 | -74.772 | 10 |
|  | Rio Grande Nature Center State Park | New Mexico | USA | 35.131 | -106.683 | 13 |
|  | Braddock Bay | New York | USA | 43.323 | -77.722 | 11 |
|  | Klamath Falls | Oregon | USA | 42.248 | -122.234 | 10 |
|  | Red Oak Trail | Texas | USA | 33.152 | -96.600 | 2 |
|  | Eau Claire | Wisconsin | USA | 44.814 | -91.277 | 13 |
| Wintering | Federal District | Distrito Federal | Mexico | 19.308 | -99.188 | 6 |
|  | Gin Creek | Arkansas | USA | 35.186 | -94.079 | 15 |
|  | Arcata | California | USA | 40.891 | -124.141 | 8 |
|  | O'Neill Forebay | California | USA | 37.080 | -121.021 | 3 |
|  | Zuma | California | USA | 34.049 | -118.812 | 20 |
|  | Cibola | California | USA | 33.366 | -114.679 | 9 |
|  | Fort Bennington | Georgia | USA | 32.371 | -85.042 | 15 |
|  | Fort Bragg | North Carolina | USA | 35.174 | -79.294 | 15 |

**Table S2.** Analysis of the molecular variance (AMOVA) for mitochondrial sequence variation among six hermit thrush populations. The two clusters revealed by Bayesian analysis (AK_pen + AK_cen + BC_west) (BC_east + MB + MI + CT) explained 88.7% of the genetic variance among groups, generating the highest φ_CT_ value.

|  | df | SS | Variance component | % of variance |
| --- | --- | --- | --- | --- |
| Among groups (φ_CT_) | 1 | 230.04 | 6.47 | 88.7*** |
| Among populations (φ_SC_) | 5 | 7.42 | 0.08 | 1.1* |
| Within populations (φ_ST_) | 64 | 47.67 | 0.74 | 10.2** |

* *p* <0.01; ** *p* <0.001; *** *p* <0.0001

**Table S3.** Factor loading for morphological traits and the derived principal components. PC1 and PC2 scores based on principal components analysis of 35 adult male hermit thrushes from western and eastern BC populations.

|  | Factor Loadings | |
| --- | --- | --- |
| Variables | PC1 | PC2 |
| Wing length | .494 | .189 |
| Kipps’ distance | .276 | .717 |
| Tail length | .408 | .158 |
| Tarsus length | .350 | -.576 |
| Bill length | .272 | -.201 |
| Bill width | .371 | -.229 |
| Bill depth | .425 | -.036 |
| Variance explained | 51.6% | 16.2% |

**Figure S1**

**

**

**Figure S2**


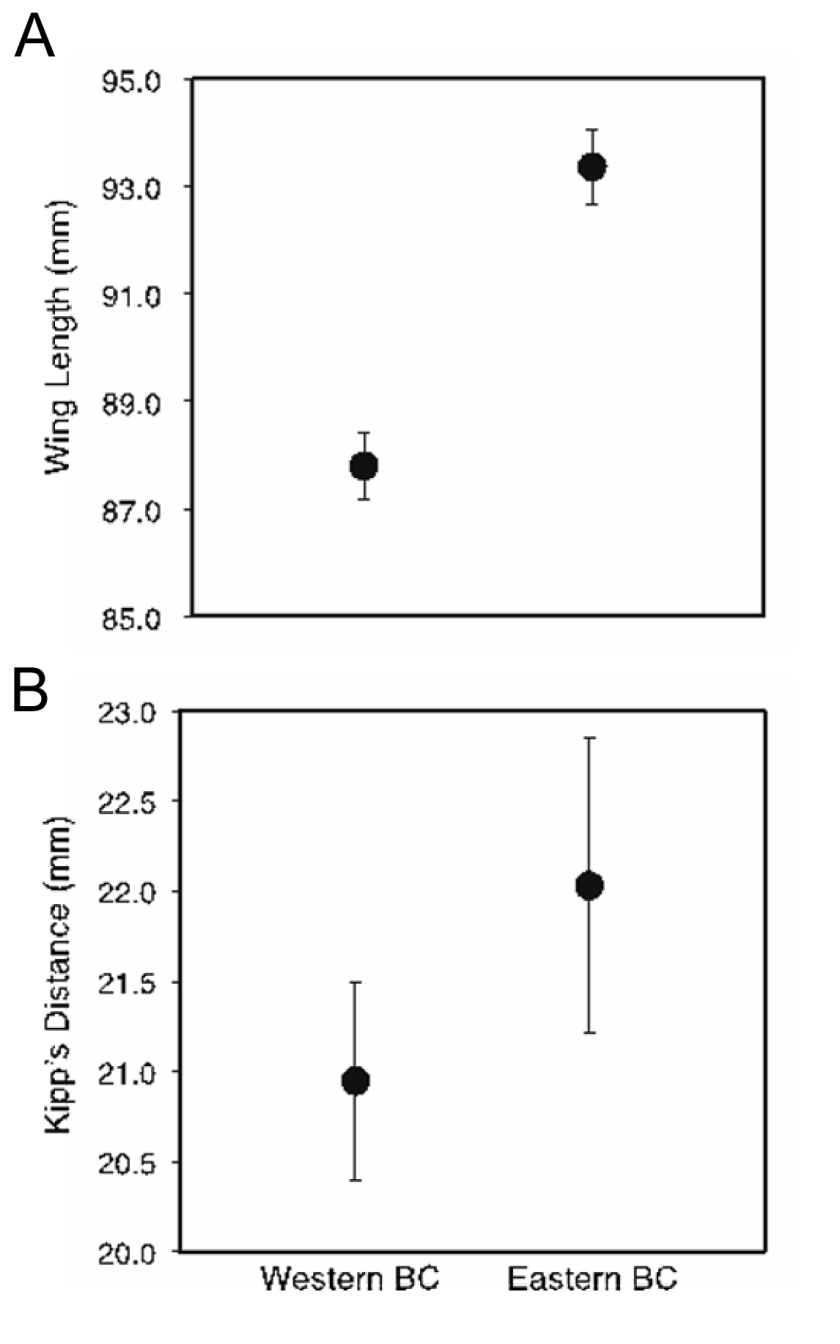


**Figure S3**


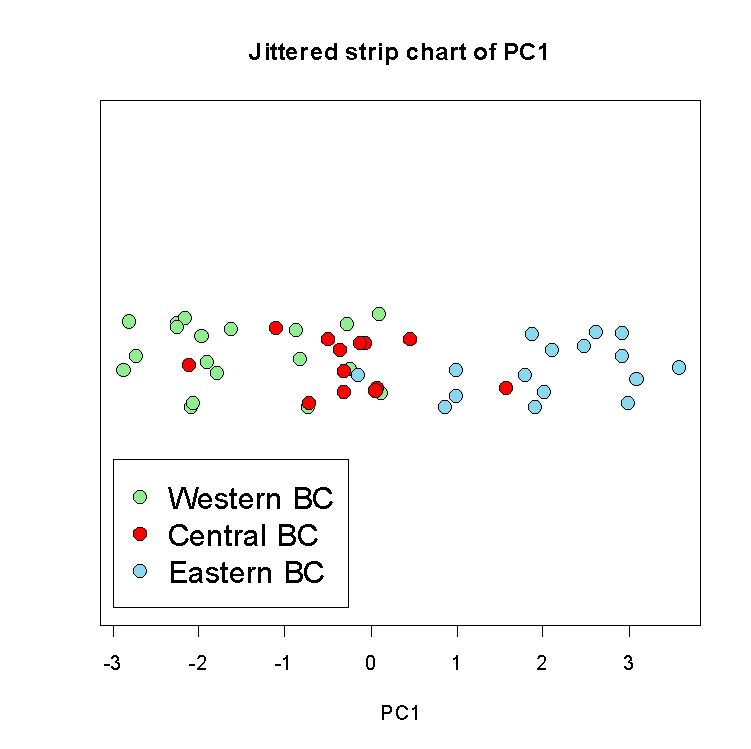

Supplement: Supplementary file 1 [file ece30004-3456-sd1.docx]
